# Qualitative study: learning from recovery: what do people who have recovered from alcohol dependence have to teach those who are still struggling?

**DOI:** 10.1192/bjo.2021.775

**Published:** 2021-06-18

**Authors:** Anju Soni, Ian Treasaden

**Affiliations:** 1 Broadmoor Hospital,West London NHS trust; 2 West London NHS trust

## Abstract

**Aims:**

The aim is to tap into user experience in the UK and to analyse what lessons can be learnt from those who have recovered from alcohol dependence to help those who are struggling including to inform the delivery of alcohol services.

**Method:**

The study was conducted in London, UK. 20 males in the age group 30–45 years were recruited. 10 of these participants had recovered from alcohol dependence and the other 10 were in treatment for alcohol dependency and diagnosed as dependent according to ICD-10 or DSM 5 criteria. In the former group, each participant had at least 2 years of complete sobriety. A semi structured questionnaire was developed and used to interview all the subjects.

Males 30-45 years were eligible as alcohol dependence is more common in this age group and purposive sampling drove the selection (i.e. if early analysis suggests the importance of a particular factor, subjects likely not to show that factor would be sampled for comparison).

Grounded analysis was the qualitative analysis method of choice and constant comparison was used, i.e., data were collected and analysed concurrently.

**Result:**

The main “families” that arose grouped around relationships in both the recovered alcoholics (RA) and continued alcoholics (CA). A successful shift required a change in the relationship to self, from feeling empty or critical towards acceptance and this shift was facilitated by being accepted and respected by others.

Relationship as motivator to stop drinking

24% people had the insight to self-refer to voluntary organisations such as AA but 76% did so because of fear of losing either their relationship or their job.

Although 80% of recovered alcoholics had been ambivalent about coming off alcohol, the shift happened when they had a nurturing relationship elsewhere such as a key worker at the Alcoholics Anonymous.

Insight and Perception

Awareness of alcohol as an obstacle rather than a solution was key for change to occur. Although 75% people with insight into their difficulties were more successful in maintaining sobriety, insight alone without action was insufficient. Moreover, action was possible without insight. Fear of death alone was a sufficient motivator.

**Conclusion:**

Difference between support systems

As a result of comparing those patients with alcohol dependence who responded well to treatment compared to those who were very recalcitrant to treatment important characteristics of an effective service have been identified. It was clear that the quality of services offered to those with alcohol dependence who attended A&E departments could be improved by offering more time for the initial assessments and adopting a more individualistic approach for each patient.

Training sessions to the A&E staff about the differences required in management between those with alcohol dependence who are motivated to abstain compared to those who will only be able to reduce consumption should be offered rather than mere blanket exhortations to abstain from alcohol consumption. The importance of behavioural avoidance of situations where alcohol is excessively consumed is more helpful in terms of eventual outcome.

The A&E staff should be encouraged to employ individuals from Alcoholics Anonymous in their department as early involvement with AA improves engagement and outcome can greatly improve engagement with treatment programs subsequently and lead to significantly better outcomes.

